# The therapeutic potential of colchicine in oncology studies: from structural modification and targeted delivery to clinical management

**DOI:** 10.3389/fphar.2026.1779469

**Published:** 2026-04-17

**Authors:** Pengyu Jin, Siyuan Wu, Haiping Yao, Yiran Ni, Ziang Yuan, Aidi Che, Lijuan Tian, Junming Li

**Affiliations:** 1 The First College of Clinical Medical Sciences, China Three Gorges University, Yichang, Hubei, China; 2 Institute of Organ Fibrosis and Targeted Drug Delivery, China Three Gorges University, Yichang, Hubei, China; 3 Yichang Central People’s Hospital, Yichang, Hubei, China

**Keywords:** anti-tumor activity, colchicine, drug delivery systems, microtubule-targeting agents, structural modification

## Abstract

Colchicine, a classical microtubule-targeting agent (MTA), exhibits remarkable anti-tumor potential, but its clinical translation is severely restricted by a narrow therapeutic window, systemic off-target toxicity, and complex pharmacokinetic vulnerabilities. Driven by the objective of “toxicity reduction and efficacy enhancement,” this review comprehensively summarizes colchicine’s multidimensional translational research. First, precise chemical modifications of its tricyclic scaffold and multi-site synergistic strategies are analyzed, exploring structure-activity relationship (SAR) mechanisms in enhancing target affinity and reversing multidrug resistance (MDR). Second, precision delivery systems—including prodrug-anchored liposomes, stimuli-responsive smart nanocarriers, inorganic mesoporous platforms, and photodynamic co-delivery—are evaluated, demonstrating their capacity to overcome biophysical barriers, reshape spatiotemporal distribution, and mediate immune synergy. Furthermore, the risks of drug-drug interactions (DDIs) and pharmacodynamic synergy arising from colchicine’s cytochrome P450 3A4 (CYP3A4)/P-glycoprotein (P-gp) dual-substrate nature in complex oncology regimens are dissected. To address this, a clinical management framework integrating ultrasensitive trace sensing and macroscopic pharmacovigilance is proposed. Finally, current translational gaps are critically confronted, notably the over-reliance on the enhanced permeability and retention (EPR) effect and the *in vitro*-*in vivo* druggability disconnect. Breakthrough paradigms like targeted protein degradation (TPD, e.g., PROTACs) and dynamic responsive carriers are subsequently proposed to guide the future precision clinical innovation of colchicine therapeutics.

## 1 Introduction

**FIGURE 1:**
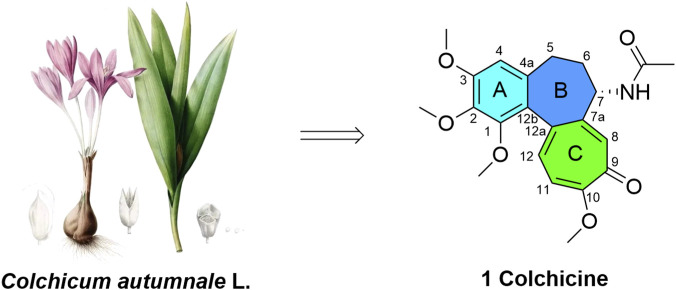
Chemical structure of colchicine.

Colchicine, a classical natural alkaloid derived from *Colchicum autumnale* L., is a highly representative member of the microtubule-targeting agents (MTAs) family (Figure 1) (Nerlekar et al., 2014; Wang et al., 2023). Long established clinically as a first-line anti-inflammatory treatment for gout and familial Mediterranean fever, its pharmacological potential as a tubulin polymerization inhibitor in oncology is currently undergoing rigorous re-evaluation by the academic community (Food and Drug Administration, 2015). Distinct from traditional chemotherapeutics that target taxane or vinca alkaloid sites, colchicine binds specifically to the β-tubulin subunit near the α/β heterodimer interface and the α-subunit’s GTP *N-*site. Through steric clash, it prevents free tubulin from transitioning from a curved to a straight conformation. This strongly inhibits microtubule polymerization, ultimately triggering mitotic arrest, spindle abnormalities, G2/M phase cell cycle arrest, and apoptosis (Wang et al., 2023).

Despite exhibiting potent *in vitro* anti-tumor activity, colchicine’s clinical utility is severely hindered by its lack of tumor-tissue selectivity. Microtubules are core cytoskeletal proteins essential for maintaining cell morphology, intracellular transport, and cell division across all normal tissues. Consequently, colchicine inevitably damages healthy, microtubule-dependent cells while inhibiting tumor proliferation. This “double-edged sword” effect remains the greatest obstacle to its oncological application (Dubey et al., 2017).

Clinically, this non-selective mechanism triggers severe target-organ toxicities. Gastrointestinal reactions are the most common early manifestations—presenting as nausea, vomiting, and diarrhea—primarily stemming from colchicine’s interference with the enterohepatic circulation and metabolic balance of bile acids (Shi et al., 2024). Furthermore, because bone marrow cells proliferate rapidly, microtubule disruption frequently causes significant myelosuppression, manifested as pancytopenia and leukopenia (Finkelstein et al., 2010). Impaired microtubule dynamics also severely interfere with neuronal axonal transport, inducing peripheral neuropathy characterized by paresthesia, while simultaneously causing abnormal autophagosome accumulation in muscle tissue, triggering myotoxicity (Liu et al., 2024; Ezzo and Etienne-Manneville, 2025). Finally, oxidative stress induced during metabolism and the disruption of tubular microtubule networks further exacerbate risks for patients with hepatic and renal impairment (Huang et al., 2024; Solak et al., 2017). Due to this exceptionally high systemic toxicity and narrow therapeutic index, neither colchicine nor any other colchicine-binding site (CBS) inhibitors have yet been approved as anti-tumor agents (Wang et al., 2023).

To address this clinical translational bottleneck, this review critically evaluates the latest advancements across the entire colchicine research continuum—from basic laboratory studies to clinical management. First, structural modification strategies are analyzed, exploring how medicinal chemistry can enhance the drug’s targeted affinity and selectivity. Subsequently, formulation innovations based on novel delivery systems designed to mitigate systemic toxicity through precision targeting are summarized. Finally, a detailed assessment of drug-drug interactions within complex, combination oncology regimens is provided. Ultimately, this review aims to provide robust, evidence-based support for the safe and effective application of colchicine in modern precision oncology.

## 2 Structural modification

Colchicine’s classical 6-7-7 tricyclic scaffold (rings A, B, and C) dictates its spatial fit and pharmacological activity at the colchicine binding site (CBS) on tubulin. However, its severe systemic toxicity and lack of tumor specificity make precise chemical modification of this core essential for broadening the therapeutic window (Ghawanmeh et al., 2020; Rubicondo et al., 2025). This section comprehensively reviews recent single-, dual-, and triple-site modifications across all three rings, systematically exploring the structure-activity relationships (SARs) designed to achieve “multidimensional synergy” between efficacy and safety, and detailing how these precise chemical alterations molecularly reshape anti-tumor activity and selectivity.

### 2.1 Single-site modification

#### 2.1.1 Ring A modification

The C1, C2, and C3 methoxy groups (-OCH_3_) on the A-ring crucially “anchor” the molecule within the tubulin binding pocket. Specifically, the C2 and C3 methoxy groups are vital for maintaining high affinity; their removal plummets the equilibrium binding constant by approximately 50%, severely attenuating inhibitory activity (Rubicondo et al., 2025). Biologically, A-ring demethylated metabolites can induce CYP3A expression, which helps mitigate *in vivo* hepatotoxicity (toxicity reduction magnitude: 3-OCH_3_ > 2-OCH_3_ > 1-OCH_3_). However, achieving highly specific C1–C3 selective demethylation synthetically remains challenging and often destabilizes the molecule’s core pharmacophore (Ghawanmeh et al., 2020).

**FIGURE 2:**
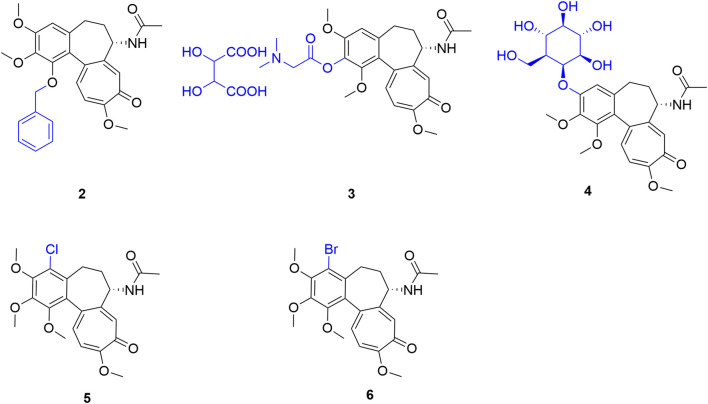
Representative compounds with modifications on the A-ring of colchicine.

Due to the methoxy groups’ vital role in colchicine’s active conformation, early A-ring optimizations prioritized isostere replacement and side-chain functionalization. Evaluating C1 modifications in a 3D model, Bartusik et al. observed that C1 demethylation sharply diminished anti-tumor activity. Conversely, introducing a bulky benzyloxy group (Figure 2, compound 2) restored it, achieving an IC_50_ of 13 ± 1 nM against human T-lymphoblastoid cells—three times the parent compound’s efficacy. However, these modifications remain in preliminary screening, lacking normal cell toxicity data (Bartusik et al., 2009). Exploring C2 modifications, Nakagawa-Goto et al. introduced polar functional groups. Although this generally attenuated *in vitro* anti-tumor activity to varying degrees, salt-formation strategies (e.g., compound 3) substantially improved aqueous solubility (>20 mg/0.1 mL). This highlights the C2 position’s potential for tuning colchicine’s physicochemical properties, particularly for enhancing druggability-related solubility (Nakagawa-Goto et al., 2005). Conversely, studies exclusively targeting the C3 position remain scarce. Exploratory work by Zarev et al. yielded a C3-glucosylated derivative (compound 4), though systematic pharmacological evaluations of this product are currently lacking (Zarev et al., 2017).

**FIGURE 3:**
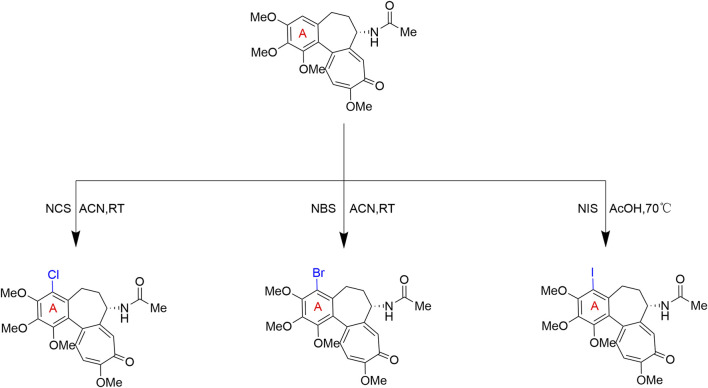
Specific halogenation reaction route at the C4 position of colchicine’s A-ring: Utilizing NCS, NBS, or NIS under specific conditions to generate C4-halogenated derivatives.

Given the sensitivity of the C1-C3 positions, researchers have shifted to the C4 position, which offers favorable spatial tolerance within the tubulin binding pocket without hindering core binding, making it advantageous for regulating tumor selectivity. Inspired by Gloryamine A, Yasobu et al. synthesized various C4-substituted (halogen, hydroxyl, carboxyl, amino, aldehyde, etc.) colchicine derivatives. Screening revealed that only C4-halogenated derivatives—obtainable via a single-step reaction (Figure 3)—exhibited potent anti-tumor activity. *In vivo*, 4-chlorocolchicine (compound 5, 5 mg/kg) achieved 68.4% tumor inhibition with zero mortality in an HCT116 xenograft model, vastly outperforming the parent colchicine (1 mg/kg), which yielded only 32.8% inhibition and 40% mortality. Furthermore, exploiting high tumoral Cathepsin B expression, a prodrug system for these C4-derivatives successfully increased the selectivity index (SI) from 0.9 to 1.4 to 1.7 (Yasobu et al., 2011). Supporting this strategy, Majcher et al. demonstrated that all C4-halogenated derivatives possessed an SI > 1, indicating significantly higher efficacy against tumor cells than toxicity toward normal BALB/3T3 cells. Molecular docking further revealed that 4-chloro (compound 5) and 4-bromo (compound 6) derivatives had stronger binding energies to βI-tubulin (−8.330 and −8.400 kcal/mol, respectively) than colchicine (−8.090 kcal/mol). Since C4-halogenation specifically enhances affinity for the βI-tubulin isotype—which is highly expressed in solid tumors like A549 (71.9%)—this interaction mechanism provides a robust structural basis for the selective eradication of cancer cells (Majcher et al., 2018a).

#### 2.1.2 Ring B modification

While the B-ring is not strictly essential for high-affinity tubulin binding, SAR studies reveal it significantly regulates biophysical properties, including binding kinetics and activation energy. Consequently, the C7 position serves as the primary modification site on this ring. Strategies targeting C7 aim to endow the molecule with novel pharmacological properties while preserving its core anti-microtubule activity (Ghawanmeh et al., 2020).

**FIGURE 4:**
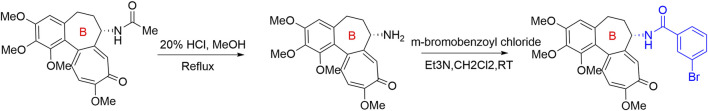
*N-*acylation modification route at the C7 position of colchicine’s B-ring: Natural colchicine undergoes acidic hydrolysis to form an *N-*deacetyl intermediate, which is then coupled with functionalized acid chlorides.

**FIGURE 5:**
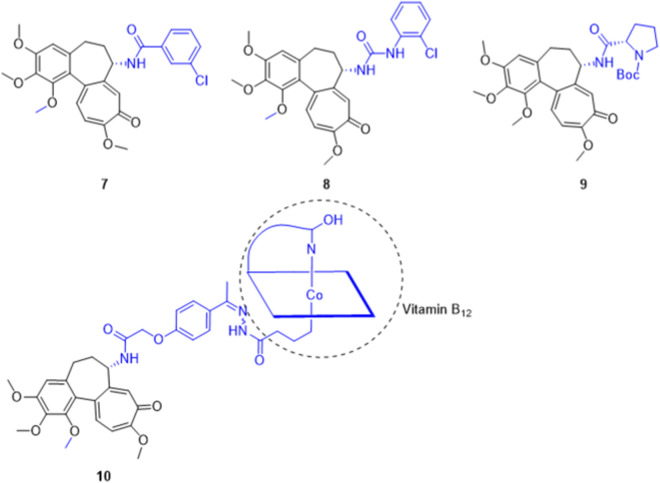
Representative compounds with modifications on the B-ring of colchicine.

The C7 side chain significantly influences colchicine’s gene-regulatory capabilities. Via an *N-*acylation route (Figure 4), Marzo-Mas et al. replaced the natural acetamido group with *N-*haloacetyl (e.g., fluoroacetyl, chloroacetyl) or *N-*haloaroyl (e.g., *m*-chlorobenzoyl, *m*-bromobenzoyl) substituents, creating derivatives that retained microtubule inhibition while profoundly downregulating tumor-associated genes (c-Myc, hTERT, and VEGF). For instance, at just 1.5 nM, the *m*-chlorobenzoyl derivative 7 (Figure 5) suppressed these genes to 20%, 59%, and 30% of control levels, respectively—an effect unachieved by 10 nM parent colchicine. Notably, this 1.5 nM concentration is far below the 20 nM antimitotic dose required for G2/M arrest, demonstrating that C7 modifications enable gene-network intervention at non-cytotoxic doses (Marzo-Mas et al., 2018). Similarly, Blasco et al. synthesized C7 urea derivatives. Their o-chlorophenylurea derivative 8 (Figure 5) exhibited an IC_50_ of 0.80 nM against HT-29 cells (colchicine: 13 nM) and downregulated c-Myc at 0.5 nM, confirming the broad utility of C7 urea/amide modifications in enhancing targeted gene regulation (Blasco et al., 2018).

To further improve the drug’s therapeutic index, Marzo-Mas’s team introduced *L*- or *D*-amino acid residues at the C7 amino group of deacetylcolchicine. The results demonstrated that the Boc-*L*-proline derivative (Figure 5, compound 9) maintained nanomolar anti-tumor activity (IC_50_) and potent gene-downregulating capability while significantly improving its safety profile. Animal experiments showed that the acute toxicity (LD_50_ > 100 mg/kg) of this derivative was reduced by approximately 40-fold compared to the parent colchicine (2.5 mg/kg), effectively broadening the drug’s therapeutic window (Marzo-Mas et al., 2021).

Furthermore, based on the strong spatial tolerance of the C7 position, exploiting the specific metabolic demands of tumor cells for active targeted delivery has become another major advantage of modification at this site. Bagnato et al. replaced the C7 acetamido group with a *p*-acetylphenoxyacetamide linker, conjugating cobalamin (vitamin B_12_) via an acid-sensitive hydrazone bond to create a targeted conjugate (compound 10). This exploits overexpressed tumoral cobalamin receptors for active cellular uptake, achieving nanomolar cytotoxicity (LC_50_: 43–302 nM) against SK-BR-3, SK-N-MC, and B16-F1 cell lines. Crucially, the pH-sensitive hydrazone bond ensures systemic stability (half-life >7 days at neutral pH) but triggers rapid drug release (half-life: 138 min) within the acidic lysosomal microenvironment (pH 4.5). This smart-release mechanism maximizes tumor-specific drug accumulation while effectively minimizing off-target toxicity to normal tissues (Bagnato et al., 2004).

#### 2.1.3 Ring C modification

The C10 methoxy group on the C-ring (a seven-membered tropolone ring) is crucial for maintaining the molecule’s chemical stability and anti-tumor proliferative activity, making it one of the core targets for colchicine structural optimization (Rubicondo et al., 2025).

**FIGURE 6:**
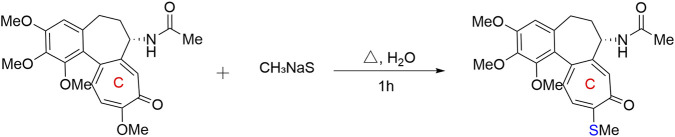
Mild thio-modification route at the C10 position of colchicine’s C-ring: Substitution of the C10-methoxy group with sodium methanethiolate under aqueous heating conditions.

**FIGURE 7:**
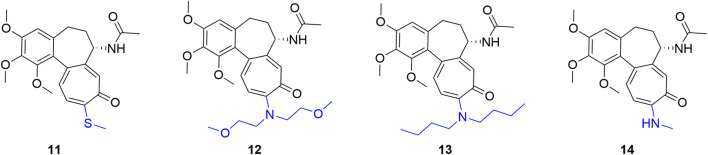
Representative compounds with modifications on the C-ring of colchicine.

Targeting the C10 position, Kurek et al. developed a green thio-modification strategy. By utilizing water-soluble sodium alkylthiolate salts (e.g., NaSCH_3_, NaSC_2_H_5_) in aqueous systems, they selectively replaced the C10 methoxy group with an alkylthio group (–SR) (Figure 6). This mild approach achieved 72%–95% yields within 1–48 h, successfully overcoming the toxic and harsh conditions of traditional methods. Pharmacological evaluations showed that the *in vitro* cytotoxicity of these derivatives was superior to the positive control drug doxorubicin. SAR analysis indicated a significant downward trend in the compound’s cytotoxicity as the length of the C10 alkylthio side chain increased (methyl → ethyl → n-propyl → isopropyl → n-butyl). Consequently, 10-thiomethylcolchicine (Figure 7, compound 11), featuring the shortest side chain, exhibited the most potent activity. It achieved an IC_50_ of 4.2 nM against DLD-1 cells (colchicine: 43.0 nM), representing a two-order-of-magnitude improvement over doxorubicin (510.6 nM) (Kurek et al., 2014).

Apart from thio-modification, amino substitution at the C10 position is another important optimization pathway. Huczyński et al. synthesized 10 such derivatives, revealing that smaller steric volume, lower polarity, or specific oxygen-containing groups enhanced anti-cancer efficacy and selectivity. For instance, compound 12 had an IC_50_ of 9 nM against HL-60 cells, exhibiting an inhibitory efficacy several times greater than that of the clinical first-line chemotherapy drugs cisplatin and doxorubicin; meanwhile, the SI of compound 13 for the LoVo/DX resistant strain reached 7.63, far higher than colchicine’s 0.18, demonstrating lower toxic side effects on normal cells (Huczynski et al., 2015).

Exploring C10 modifications to overcome multidrug resistance (MDR) and metabolic vulnerabilities, Yang et al. demonstrated that 10-methylaminocolchicine (compound 14) achieved a GI_50_ of 5.0 ± 3.2 nM in K562 cells, roughly doubling the parent colchicine’s efficacy (10.7 ± 5.1 nM). Mechanistically, colchicine’s natural C10 methoxy group is highly susceptible to CYP3A4-mediated demethylation, yielding the inactive and potentially toxic colchiceine. The C10 methylamino modification effectively circumvents this metabolic defect. Crucially, compound 14 exhibited exceptional cross-resistance reversal in paclitaxel-resistant A549/T cells (GI_50_: 23.64 nM), vastly outperforming colchicine (1083.40 nM) and paclitaxel (4,755 nM), thereby slashing the resistance index (RI) from 28.25 to 3.44. Molecular docking indicated that the C10 methylamino group forms stable hydrogen bonds with the Gln725 residue of P-glycoprotein (P-gp), blocking its efflux function. *In vivo*, a mere 0.5 mg/kg dose of compound 14 yielded a 64.2% tumor inhibition rate in an LL/2 mouse model, significantly surpassing 10 mg/kg paclitaxel and highlighting this modification’s potential for enhancing the overall therapeutic index (Yang et al., 2023).

### 2.2 Dual-site modification

Multi-site modification has recently emerged as a crucial strategy to comprehensively enhance colchicine’s anti-tumor activity and targeting selectivity while mitigating systemic toxicity. Its core logic relies on exploiting the synergistic effects of multiple modification sites to optimize β-tubulin binding affinity, improve pharmacokinetic profiles, and boost tumor-specific recognition. This approach effectively overcomes single-site limitations, offering a robust pathway to substantially expand the drug’s therapeutic index (Rubicondo et al., 2025). In this evolutionary process, dual-site modification represents the most fundamental and widely explored multidimensional approach. Current research primarily emphasizes the synergistic optimization of “B-ring + C-ring” and “A-ring + C-ring” combinations.

#### 2.2.1 B-Ring and C-Ring synergy (C7 + C10 positions)

In B/C ring synergistic modifications, replacing the C10 methoxy group with a more metabolically stable methylamino (–NHCH_3_) or methylthio (–SCH_3_) group typically serves as the foundational scaffold optimization. Building upon this premise, structurally diversifying the B-ring C7 side chain has demonstrated tremendous potential for simultaneously reducing toxicity and enhancing efficacy.

**FIGURE 8:**
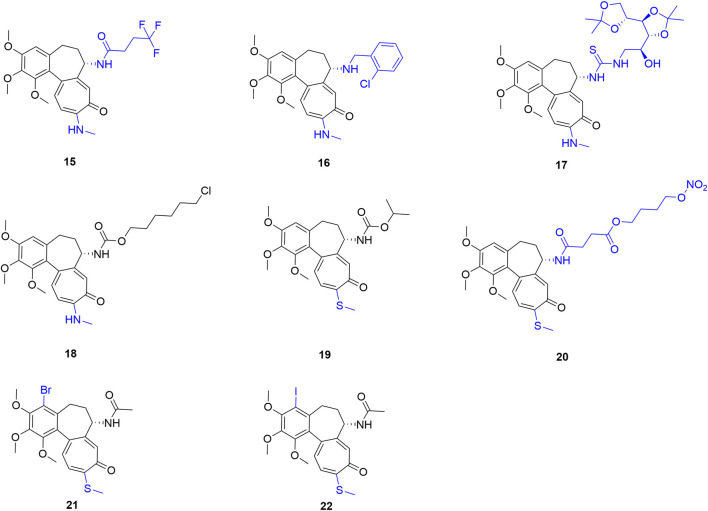
Representative compounds of dual-site synergistic modifications of colchicine.

Systematically exploring dual-site modifications, the Krzywik team synthesized C7 amide/sulfonamide and C10 methylamino derivatives. Notably, compound 15 (Figure 8)—featuring a C7 4,4,4-trifluorobutyramide and C10 *N*-methylamino group—potently inhibited A549, MCF-7, and LoVo cells (IC_50_: 10.4–14.6 nM; colchicine: 17.5–115.3 nM) with a SI up to 7.8 (Krzywik et al., 2020a). To enhance metabolic stability, they subsequently replaced the C7 acetamido with secondary amines (e.g., benzylamino, alkylamino). The standout molecule, compound 16, achieved remarkable IC_50_ values of 0.1 nM and 1.6 nM against LoVo and its resistant LoVo/DX subline, respectively. Its efficacy against the resistant strain outperformed colchicine by ∼440-fold, while an exceptional SI of 93.7 demonstrated extraordinarily high tumor-killing specificity (Krzywik et al., 2020b).

To broaden the SAR space, researchers extended the C7 side chain to urea, thiourea, and carbamate systems. Introducing a protected glucitol thiourea (Figure 8, compound 17) yielded excellent selectivity (SI = 29.3 against MCF-7 cells). Conversely, guanidino substitution abolished activity, confirming that carbonyl or thiocarbonyl groups are essential for microtubule binding. Moreover, this thiourea series effectively reversed MDR, achieving a RI as low as 4 (doxorubicin: 69; colchicine: 250) (Krzywik et al., 2021a). Their subsequent C7 carbamate/thiocarbamate series performed equally well. Notably, the C7 6-chlorohexyloxycarboxamide derivative 18 exhibited extraordinary IC_50_ values of 0.1 ± 0.01 nM against LoVo (SI = 87.1) and 1.2 ± 0.4 nM against A549 cells—an *in vitro* efficacy ∼4,100-fold greater than the clinical drug cisplatin (Krzywik et al., 2021b).

Regarding the synergy with C10 thio-modification, Majcher et al. explored the combination of a C10 methylthio group with C7 carbamate substitution. *In vitro* evaluations revealed that C7 carbamates bearing saturated or fluoroalkyl chains exhibited broad-spectrum anti-cancer activity and strong tumor selectivity. Notably, compound 19 proved most potent, achieving an IC_50_ of 9 nM against MCF-7 cells and an SI of 16.28 (over 8-fold higher than colchicine’s 1.9). Further SAR analysis indicated that moderately increasing C7 lipophilicity enhanced efficacy, whereas highly polar hydroxyls or long flexible ether chains diminished it. These findings provide clear structural guidelines for optimizing the physicochemical properties and spatial conformation of the B-ring side chain (Majcher et al., 2018b).

Beyond optimizing binding affinity, Shen et al. exploited functional group synergy by combining a C10 methylamino modification with a C7 nitric oxide (NO) donor (e.g., nitrate ester, –ONO_2_), constructing chimeric molecules with dual killing mechanisms. Against cell lines like A2780, A549, BEL7402, and MCF-7, targeted NO release significantly amplified cytotoxicity. Notably, compound 20 achieved an IC_50_ of 8 nM against MCF-7 cells—a ∼10-fold improvement over colchicine (84 nM). This successfully validates the “microtubule inhibition + NO biological effect” superposition strategy for amplifying anti-tumor efficacy (Shen et al., 2011).

#### 2.2.2 A-Ring and C-Ring synergy (C4 + C10 positions)

Exploring A- and C-ring synergy, Majcher et al. combined C4 halogenation with C10 thiomethyl substitution to synthesize novel thiocolchicine derivatives. *In vitro*, the 4-bromo-10-thiomethyl derivative 21 (Figure 8) demonstrated excellent broad-spectrum efficacy, achieving IC_50_ values of 10, 15, and 14 nM against A549, MCF-7, and LoVo cells, respectively—a 7.7- to 14.9-fold improvement over colchicine. Furthermore, compound 21 effectively reversed MDR, slashing the RI in LoVo/DX cells to 9.6 (colchicine: 24.5). Within this series, 4-iodo-thiocolchicine (compound 22), featuring a bulkier A-ring iodine, exhibited the highest targeting selectivity for LoVo cells (SI = 16.4). These findings confirm that while C10 thio-modifications scaffold high anti-microtubule efficacy, C4 halogens are crucial for fine-tuning tumor-specific recognition and broadening the safety window (Majcher et al., 2018a).

### 2.3 Triple-site synergistic modification (Ring A + Ring B + Ring C)

**FIGURE 9:**
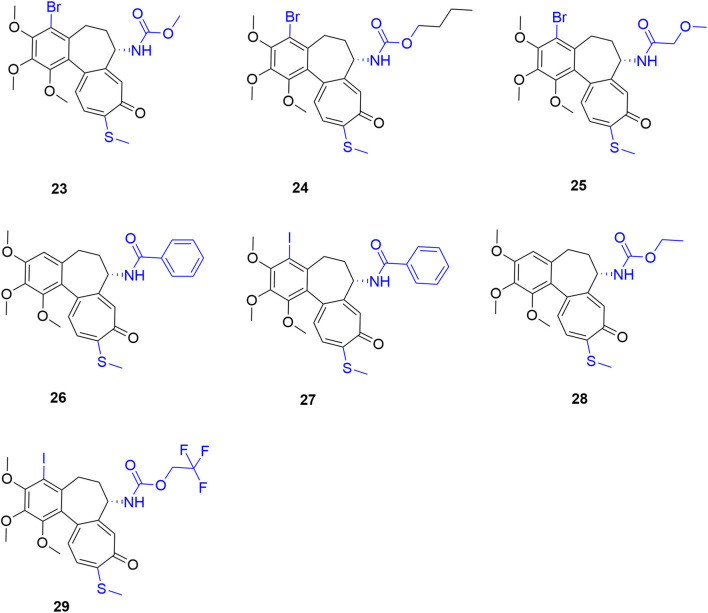
Representative compounds of triple-site synergistic modification of colchicine. Dual-site analogs (compounds 26 and 28) are included for SAR comparison.

Simultaneous precise alteration of the A, B, and C rings—specifically the “C4 halogenation + C7 functionalization + C10 thio-modification” strategy—deeply remodels colchicine’s pharmacological and thermodynamic profile. Pioneering this, Majcher et al. pioneered triple-site synergistic modifications combining C4 bromination, C7 carbamoylation, and C10 thio-modification. This strategy yielded derivatives with potent nanomolar cytotoxicity. Notably, derivative 23 (Figure 9) exhibited broad-spectrum anti-tumor activity with IC_50_ values as low as 7 nM. Furthermore, derivative 24, which incorporates an n-butoxycarbonyl group, demonstrated excellent efficacy against the resistant LoVo/DX strain, sharply reducing the RI from 15.65 (colchicine) to 1.5. Concurrently, off-target toxicity was substantially mitigated; the SI of compound 23 for A549 cells increased to 6.60 (colchicine: 1.11), validating the capacity of triple-site modifications to broaden the therapeutic safety window (Majcher et al., 2018c).

Following this approach, Klejborowska et al. designed derivatives combining C4 bromination (to enhance target affinity), a C10 methylthio group (for stability), and various C7 amides (to reduce toxicity and boost efficacy). Notably, compound 25 achieved an IC_50_ of 5.3 nM against primary acute lymphoblastic leukemia (ALL-5) cells (colchicine: 8.6 nM), while maintaining excellent 10–11 nM activity across multiple solid tumor lines. However, despite moderate selectivity (SI > 2) for ALL-5 and LoVo cells in some compounds, the overall average selectivity of this triple-modified series decreased relative to their precursors. This indicates that complex functional group combinations require precise fine-tuning to avoid narrowing the therapeutic window (Klejborowska et al., 2019).

To determine the A-ring halogen’s pharmacodynamic contribution, the team compared “dual-site (C7 amide + C10 thio)” and “triple-site (C4 iodo + C7 amide + C10 thio)” derivatives. This revealed a distinct targeting divergence: dual-site molecules preferred ALL-5 cells, whereas triple-site molecules favored LoVo colon cancer cells. Specifically, against ALL-5, the dual-site compound 26 (Figure 9) significantly outperformed the triple-site compound 27 (IC_50_: 5.7 ± 1.9 nM vs. 25.4 ± 0.8 nM). Conversely, against LoVo cells, compound 27 slightly edged out compound 26 (7.2 ± 0.1 nM vs. 8.5 ± 1.3 nM). Molecular docking thermodynamically corroborated these findings: the C4-iodinated triple-site molecule yielded a superior binding energy (−9.30 kcal/mol) compared to the dual-site analog (−8.70 kcal/mol), confirming that A-ring halogenation crucially enhances target affinity (Klejborowska et al., 2021).

Urbaniak et al. corroborated this “targeting preference” using primary tumor models, evaluating C7 carbamate derivatives across ALL-5 and two clinical breast cancer subtypes (ER^+^/PR^+^/HER2^-^ IDCG3, and triple-negative MC). Dual-site derivatives (C7 + C10) demonstrated outstanding absolute efficacy (ALL-5 IC_50_: 1.1–1.9 nM) and unique synergy. Notably, combined with the microtubule destabilizer vincristine (VCR), compound 28 exhibited potent synergy (combination index, CI = 0.58)—whereas colchicine showed antagonism—indicating a critical shift in substrate recognition. Conversely, while C4-halogenated triple-site molecules did not surpass dual-site analogs against leukemia, they exhibited superior broad-spectrum activity across the NCI-60 panel (e.g., non-small cell lung, colon, CNS, and prostate cancers). Crucially, they offered a broader safety window; compound 29 achieved an SI of 5.2 against IDCG3 cells (colchicine: 1.5), demonstrating lower toxicity toward normal mammary epithelial cells (MCF-10A). Ultimately, dual- and triple-site modifications are not mere linear “superpositions” of efficacy. Instead, they form highly complementary SAR profiles across killing efficacy, combination potential, and lineage selectivity. This provides a structural and translational foundation for differentiated precision oncology (e.g., intensive combination therapy for leukemia vs. precision monotherapy for specific breast cancers) (Urbaniak et al., 2020).

## 3 Tumor-targeted delivery and formulation optimization: translational strategies to break through the therapeutic window of colchicine

Despite significant progress in chemical modifications to enhance microtubule affinity, free colchicine derivatives still face systemic druggability bottlenecks, including poor water solubility, short half-lives, and severe off-target toxicity. To fundamentally alter their pharmacokinetic (PK) profiles and enable lesion-specific accumulation, modern nanomedicine and targeted delivery approaches are essential (Lei et al., 2024). This section explores innovative formulation designs aimed at breaking through colchicine’s extremely narrow therapeutic window.

### 3.1 Prodrug-based liposomal encapsulation strategy

As a potent vascular disrupting agent (VDA), colchicine’s moderate lipophilicity (logP ∼ 1.0) causes rapid transmembrane leakage from traditional liposomes (reaching 100% release within 24 h). This premature systemic burst release curtails tumor accumulation via the enhanced permeability and retention (EPR) effect, exacerbating systemic toxicity.

**FIGURE 10:**
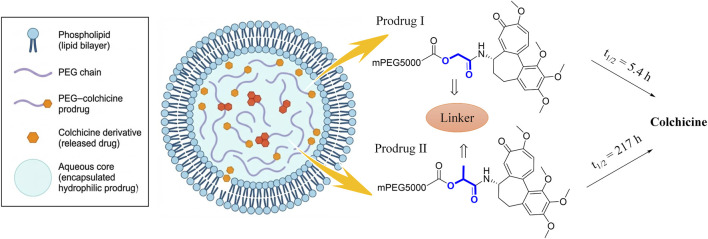
Long-circulating liposomal delivery model based on PEGylated prodrugs and their *in vitro* hydrolysis kinetics.

To overcome this, Crielaard et al. developed a liposomal delivery system using hydrolyzable poly (ethylene glycol) (PEG)ylated prodrugs. By attaching biodegradable linkers, these highly water-soluble prodrugs remain stably trapped within the liposome’s aqueous core (Figure 10). Their release is strictly governed by ester bond hydrolysis: Prodrug I (a primary alcohol ester with a glycolic acid linker) hydrolyzed rapidly (half-life: 5.4 h at 37 °C, pH 7.4), whereas the sterically hindered Prodrug II (a secondary alcohol ester with a lactic acid linker) hydrolyzed extremely slowly (half-life: 217 h). This elegant design prevents systemic leakage and allows precise customization of VDA release kinetics (Crielaard et al., 2012).

**FIGURE 11:**
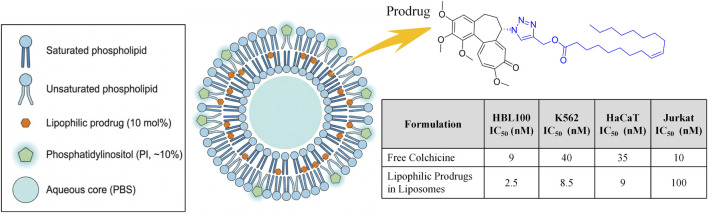
Delivery strategy of lipophilic prodrugs anchored in the lipid bilayer and their *in vitro* anti-tumor activity advantages compared to free drugs.

Alternatively, leakage can be prevented by designing lipophilic prodrugs that anchor directly into the lipid bilayer. Kuznetsova et al. synthesized fatty acid esters (e.g., palmitate, oleate) of triazole-colchicine analogues, formulating them into ∼100 nm liposomes (Figure 11). These highly lipophilic long chains firmly integrate into the fluid bilayer, achieving a 10 mol% drug loading capacity with negligible storage leakage. *In vitro*, these lipophilic prodrugs outperformed their parent compounds, relying on enhanced cellular uptake and rapid intracellular esterase cleavage. Notably, the liposomal oleate prodrug exhibited an exceptional IC_50_ of 2.5 nM against human breast epithelial cells (HBL-100), significantly surpassing the free parent analogue. This bilayer-loading strategy simplifies hydrophobic drug encapsulation, offering a robust, high-capacity platform with potent targeted-killing potential (Kuznetsova et al., 2013).

### 3.2 Local sustained release based on biodegradable microspheres

**FIGURE 12:**
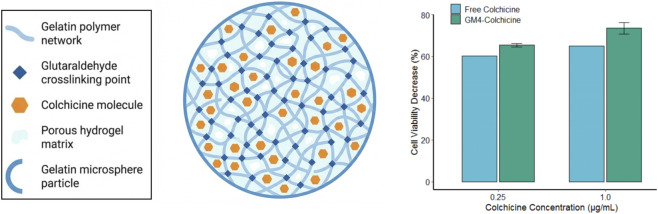
Degradable gelatin microsphere sustained-release system encapsulating colchicine and its long-acting proliferation-inhibitory effect on MCF-7 breast cancer cells.

To overcome the severe systemic toxicity of anti-tumor agents like colchicine, degradable natural polymer microspheres offer a robust solution for long-acting, localized intratumoral delivery. Muvaffak et al. prepared colchicine-loaded degradable gelatin microspheres via water-in-oil emulsion polymerization using glutaraldehyde crosslinking (Figure 12). By tuning the crosslinker concentration, the researchers precisely controlled microsphere swelling, matrix degradation, and drug release kinetics. Low crosslinking yielded high swelling and rapid degradation-release, whereas a high-density network restricted matrix expansion, producing an exceptionally slow release profile. *In vitro* evaluations highlighted the dynamic advantages of this sustained-release system: while free colchicine exerted rapid acute toxicity, surviving MCF-7 breast cancer cells recovered and resumed proliferation over a 4-day culture. Conversely, the gradual gelatin matrix degradation and continuous drug diffusion from low-crosslinking microspheres applied a sustained, stable cytotoxic pressure. Ultimately, at a 1.00 μg/mL dose, the sustained-release microspheres reduced cell viability by 74%, significantly outperforming the free drug (65%). This fully biodegradable, localized formulation maintains high peritumoral drug concentrations, substantially enhancing long-term chemotherapeutic efficacy and safety by prolonging the duration of action (Muvaffak et al., 2004).

### 3.3 Active targeted delivery mediated by receptors and biological barrier penetration

**FIGURE 13:**
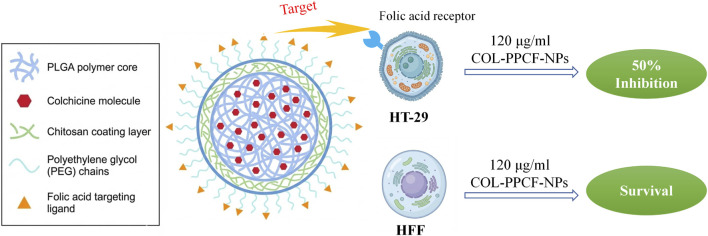
Folate receptor-mediated multifunctional polymeric nanodelivery system (COL-PPCF-NPs) and its selective killing of HT-29 colon cancer cells.

Beyond CD44, folate receptor (FR)-mediated active targeting is a crucial strategy for precision solid tumor therapy. Sadeghzadeh et al. formulated a multifunctional polymeric nanodelivery system (COL-PPCF-NPs) via a double emulsion-solvent evaporation method (Figure 13). This system features a colchicine-loaded hydrophobic PLGA core, enveloped by a chitosan (CS) and PEG biomimetic coating. This coating’s hydrophilicity and steric hindrance effectively block reticuloendothelial clearance, ensuring excellent “stealth” and structural stability. Crucially, surface-conjugated folic acid (FA) ligands precisely anchor to overexpressed FRs on HT-29 colon cancer cells, significantly enhancing intracellular accumulation via receptor-mediated endocytosis. *In vitro*, this achieved “selective toxicity”: at 120 μg/mL, it inhibited ∼50% of HT-29 cells while sparing normal human fibroblasts. Mechanistically, targeted colchicine induced S and G2/M phase arrest and robustly activated intrinsic apoptosis (upregulating Bax, Caspase-3/9, and P53). By integrating stealth evasion, targeted recognition, and selective killing, this nanoplatform offers a solid foundation for toxicity-reducing colchicine applications (Sadeghzadeh et al., 2023).

**FIGURE 14:**
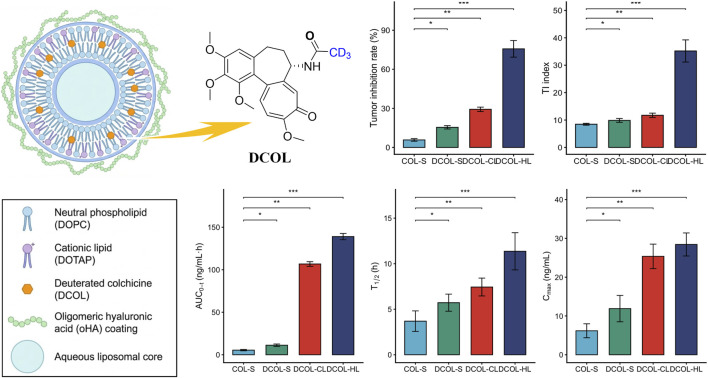
Oligomeric hyaluronic acid (oHA)-modified deuterated colchicine liposome (DCOL-HL) model and the comparison of its pharmacological and pharmacokinetic parameters with free colchicine. (Note: COL-S, Colchicine Solution; DCOL-S, Deuterated Colchicine Solution; DCOL-CL, DCOL Cationic Liposomes; DCOL-HL, oHA-DCOL Liposomes.)

To definitively conquer colchicine’s narrow therapeutic window and systemic off-target toxicity, Chen et al. innovatively integrated “chemical isotopic modification” with “active targeted nanodelivery” to develop oligomeric hyaluronic acid (oHA)-modified deuterated colchicine liposomes (DCOL-HL) (Figure 14). Molecularly, deuterium substitution at the C7 side-chain methyl group (DCOL) increased the carbon-deuterium bond cleavage activation energy. This significantly slowed hepatic metabolism, drastically reduced highly toxic deacetylated metabolites, and fundamentally mitigated hepatotoxicity. For delivery, DCOL was loaded into cationic liposomes electrostatically coated with oHA. This biomimetic oHA shell provides smart-responsive features: in circulation, it shields the positive charge and reduces protein adsorption (prolonging half-life); within the slightly acidic, hyaluronidase-rich tumor microenvironment (TME), specific oHA degradation triggers a surface charge reversal (−20 mV to +40 mV), breaking the charge barrier for deep penetration. Furthermore, oHA specifically targets overexpressed CD44 receptors on triple-negative breast cancer (4T1) cells, mediating highly efficient endocytosis. Pharmacokinetically, the targeted liposomes’ AUC_0-_t was 25.6-fold higher than the free solution. *In vivo*, DCOL-HL achieved a 75.71% tumor inhibition rate, expanding the therapeutic index (TI) from 8.46 (free drug) to 35.20. This “deuteration + targeted delivery” dual strategy completely abolished free colchicine-induced fatal liver injury while profoundly suppressing VEGF-mediated angiogenesis, *in situ* growth, and lung metastasis (Chen et al., 2023).

**FIGURE 15:**
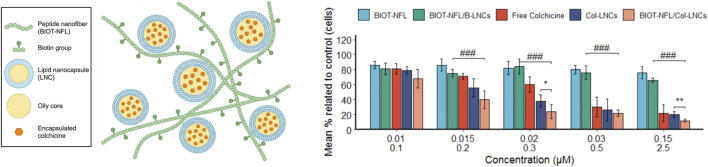
Schematic of the novel smart targeted delivery system combining colchicine-loaded lipid nanocapsules (Col-LNCs) with biotinylated cell-penetrating peptide (BIOT-NFL) and a comparison of its tumor cell proliferation inhibitory efficacy relative to free colchicine. (Note: BIOT-NFL, Biotinylated-NFL-peptide; BIOT-NFL/B-LNCs, BIOT-NFL conjugated blank lipid nanocapsules; Col-LNCs, Colchicine-loaded Lipid Nanocapsules; BIOT-NFL/Col-LNCs, BIOT-NFL conjugated colchicine-loaded lipid nanocapsules.)

Addressing the formidable clinical challenge of glioblastoma (GBM)—characterized by blood-brain barrier (BBB) obstruction and poor targeting—Alnemeh-Al Ali et al. engineered a smart delivery system combining colchicine-loaded lipid nanocapsules (Col-LNCs) with a biotinylated cell-penetrating peptide (BIOT-NFL) (Figure 15). Colchicine was encapsulated within the LNC oily core via phase inversion, followed by electrostatic surface adsorption of nanofiber-forming BIOT-NFL peptides. Remarkably, the NFL peptide inherently disrupts microtubule networks and serves as a highly specific targeting carrier for GBM cells. *In vitro*, this “peptide nanofiber-LNC” composite efficiently entered F98 rat glioblastoma cells via receptor-mediated endocytosis, triggering perinuclear colchicine release. Compared to non-targeted vesicles, this targeted system drastically dismantled the GBM microtubule network, significantly enhancing anti-proliferative efficacy while averting off-target toxicity to normal cells. This platform demonstrates the superiority of peptide ligands in augmenting liposomal internalization and introduces a highly promising translational concept for delivering colchicine across the BBB for precise CNS tumor therapy (Alnemeh-Al Ali et al., 2025).

### 3.4 Organic-inorganic hybrid mesoporous nanoplatforms and immune/genetic synergistic intervention

**FIGURE 16:**
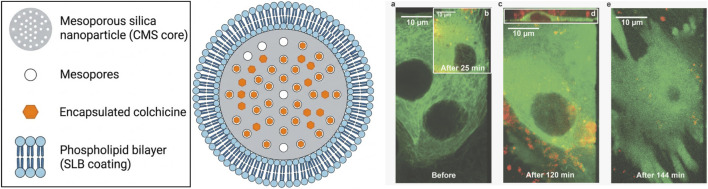
Schematic diagram of the lipid bilayer-coated mesoporous silica hybrid delivery system (SLB@CMS) and its mediated intracellular concentrated drug release effect in liver cancer cells. (Left) Schematic structure of SLB@CMS: Mesoporous silica (gray core) encapsulates colchicine (orange) internally, and the outer layer is tightly coated by an intact phospholipid bilayer (blue) to prevent premature drug leakage. (Right) Dynamic live-cell confocal imaging confirms that in the early stages of incubation, the drug-loaded particles merely attach to the cell surface without drug leakage (microtubules remain intact); subsequently, a large number of particles are endocytosed into the cell and centrally release the drug, ultimately leading to complete microtubule depolymerization and morphological disintegration and death of the cells. Reprinted with permission from Cauda et al 2010. Copyright 2010 American Chemical Society.

To balance high drug loading capacity with the prevention of premature release, Cauda et al. developed a 50 nm colloidal mesoporous silica (CMS) nanohybrid coated with a supported lipid bilayer (SLB@CMS) (Figure 16). Via solvent exchange, an intact SLB was self-assembled onto surface-aminated CMS particles. This biomimetic lipid shell ensures superior biocompatibility and colloidal stability while acting as a dense physical barrier, perfectly “sealing” guest molecules within the mesopores to prevent premature systemic leakage. *In vitro*, incubating colchicine-loaded SLB@CMS with HuH7 liver cancer cells revealed highly efficient endocytosis. Once cytoplasmic, colchicine penetrated the lipid layer for concentrated local release, rapidly inducing microtubule depolymerization and cell death. Crucially, compared to equivalent free colchicine, this endocytosis-mediated intracellular “concentrated delivery”—rather than slow extracellular passive diffusion—profoundly amplified the drug’s anti-microtubule and cytotoxic efficacy (Cauda et al., 2010).

**FIGURE 17:**
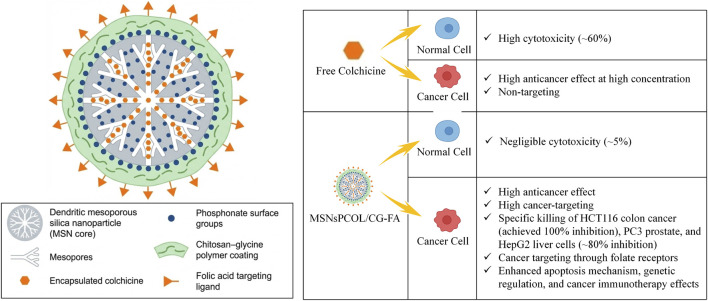
Schematic of the dendritic mesoporous silica targeted delivery platform (MSNsPCOL/CG-FA) mediating synergistic gene and immune regulation, and its pharmacological advantages compared to free colchicine.

To broaden colchicine’s multidimensional mechanisms against colon cancer, AbouAitah et al. engineered an active targeted delivery system using dendritic mesoporous silica nanoparticles (MSNsPCOL/CG-FA) (Figure 17). Initially, MSNs were surface-functionalized with phosphonate groups for highly efficient colchicine loading, then coated with a chitosan-glycine (CG) polymer conjugated to FA. This composite shell effectively controls drug release while precisely recognizing overexpressed folate receptors on solid tumors, thereby maximizing receptor-mediated endocytosis. *In vitro*, this nanosystem achieved 100% growth inhibition in HCT116 colon cancer cells but exhibited merely 5% toxicity toward normal fibroblasts (BJ1)—starkly contrasting the ∼60% toxicity of free colchicine—successfully realizing targeted “toxicity reduction and efficacy enhancement.” Mechanistically, beyond standard anti-microtubule effects (G2/M arrest and intrinsic apoptosis via Bax upregulation and cytochrome c release), this targeted platform demonstrated cutting-edge potential for gene regulation and immune intervention. Target-delivered colchicine significantly downregulated tumor-promoting long non-coding RNA (MALAT1) and microRNA (mir-205). Crucially, the formulation potently suppressed pro-angiogenic factors (Ang-2) and immune checkpoint molecules (PD-1). This research validates the superiority of inorganic porous nanomaterials for colchicine delivery and unveils the emerging translational value of colchicine nanoformulations in remodeling the tumor microenvironment to synergize with cancer immunotherapy (AbouAitah et al., 2020).

### 3.5 Multimodal combination therapy for deep penetration and drug resistance

**FIGURE 18:**
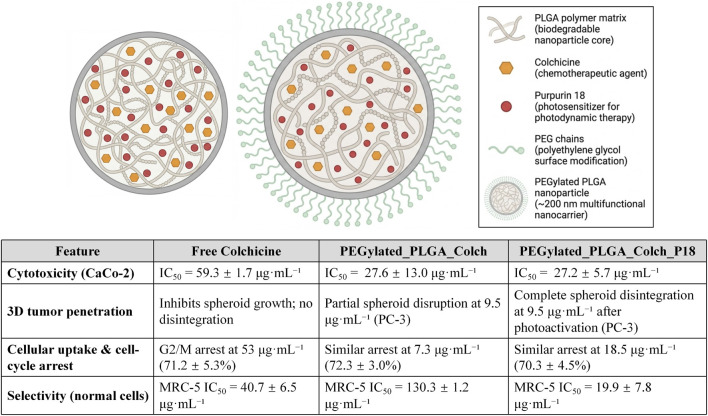
Schematic of the multimodal co-delivery nanoplatform based on PLGA and its PEGylated derivatization, and the comparison of pharmacological profiles among free colchicine, single-drug nanoparticles (PEGylated_PLGA_Colch), and dual-drug nanoparticles (PEGylated_PLGA_Colch_P18).

Beyond single-drug targeted delivery, multimodal co-delivery systems combining colchicine with secondary therapeutic modalities represent a frontier for overcoming resistance in complex solid tumors. Villela Zumaya et al. developed multifunctional poly (lactic-co-glycolic acid) (PLGA) and PEGylated-PLGA nanocarriers for the highly efficient co-encapsulation of colchicine (Colch) and the photosensitizer Purpurin 18 (P18) (Figure 18). This system ingeniously and spatiotemporally unifies colchicine’s anti-microtubule cytotoxicity with photodynamic therapy (PDT). *In vitro*, both single- and dual-drug PEGylated PLGA nanoparticles exhibited enhanced cytotoxicity against CaCo-2 colon cancer cells compared to free colchicine. Notably, the single-drug formulation (PEGylated_PLGA_Colch) significantly reduced off-target toxicity toward normal MRC-5 cells (IC_50_: 130.3 μg/mL). While the dual-drug nanoparticles (PEGylated_PLGA_Colch_P18) showed increased cytotoxicity in 2D normal cultures, their true translational breakthrough emerged in a biomimetic 3D PC-3 prostate cancer multicellular spheroid model. Driven by the synergistic photodynamic effect, photoactivated dual-drug nanoparticles achieved deep tumor penetration and complete spheroid disintegration. This profound structural destruction significantly outperformed both free colchicine and single-drug nanoparticles, which merely caused partial disruption. Ultimately, by integrating “chemo-photodynamic therapy,” this multimodal nanoplatform drastically amplifies colchicine’s anti-tumor power, offering a highly promising translational paradigm for treating complex and refractory solid tumors (Zumaya et al., 2024).

## 4 Drug interactions and clinical management recommendations for colchicine in tumor therapy scenarios

### 4.1 Pharmacokinetic vulnerability and risks of combination therapy

As an alkaloid with an extremely narrow therapeutic window, colchicine relies heavily on cytochrome P450 3A4 (CYP3A4) and the P-gp efflux transporter. Physiologically, these proteins synergistically restrict systemic exposure via first-pass metabolism and efflux. However, this “dual-substrate” nature harbors severe, potentially fatal drug-drug interaction (DDI) risks. Co-administering strong CYP3A4 or P-gp inhibitors blocks efflux and saturates metabolism, drastically reducing clearance. The resulting concentration spikes can precipitate myelosuppression and fatal multi-organ failure (Finkelstein et al., 2010).

In oncology, this PK vulnerability is amplified. Intense systemic inflammation and massive pro-inflammatory cytokine release (e.g., IL-6, TNF-α) from tumor and immune cells significantly downregulate hepatic CYP enzymes (especially CYP3A4) at the transcriptional level. Consequently, even prior to combination therapy, a tumor patient’s intrinsic colchicine clearance operates at a compromised baseline (Harvey and Morgan, 2014).

Compounding this baseline, supportive care drugs like triazole antifungals (e.g., itraconazole, a strong dual CYP3A4/P-gp inhibitor) can easily trigger accumulative toxicity. The FDA strongly mandates substantial colchicine dose reductions with such inhibitors; for patients with hepatic or renal impairment, this combination is strictly contraindicated (Hansten et al., 2023; Wang et al., 2025). Furthermore, molecularly targeted therapies (e.g., TKIs or CDK4/6 inhibitors like crizotinib and ribociclib) are categorized as ORCA Class 2 risks (avoid unless benefits clearly outweigh risks). Because these combinations cause highly variable AUC increases (54%–924%), clinical management should prioritize discontinuing colchicine or switching anti-tumor agents. If unavoidable, a 50%–75% colchicine dose reduction under strict monitoring is required, carefully balancing toxicity risks against potential treatment failure (Hansten et al., 2023).

### 4.2 Pharmacodynamic interactions: anti-tumor targeted synergy and mechanistic coupling

While PK interactions pose severe toxicity risks, rational pharmacodynamic (PD) combinations can exploit colchicine’s strategic synergistic potential. For instance, in multiple myeloma, combining colchicine with bortezomib inhibits SAE1-mediated SUMOylation and p27 nuclear export, effectively restoring cell cycle arrest via mechanistically coupled synergy (Wang et al., 2025). In hepatocellular carcinoma, co-administering colchicine with lenvatinib inhibits receptor tyrosine kinase pathways and stemness markers (NANOG), antagonizing the pro-stemness and resistance risks of kinase inhibitor monotherapy (Lin et al., 2023). Notably, these PD interactions are context-dependent: colchicine synergizes with amygdalin in osteosarcoma but exhibits antagonism in chondrosarcoma (Aydin et al., 2021). Thus, colchicine’s PD synergy relies heavily on the tumor’s specific molecular phenotype and microenvironment.

### 4.3 Therapeutic drug monitoring (TDM) and macroscopic pharmacovigilance systems

Given colchicine’s “double-edged sword” profile—high clinical benefit offset by a narrow therapeutic index and immense inter-individual PK variability—establishing robust TDM and risk warning systems is paramount. While traditional detection relies on complex chromatography, recent advances in trace sensing offer rapid detection in complex biological matrices. For example, Kui et al. engineered a molecularly imprinted photoelectrochemical sensor using a Z-scheme SnIn_4_S_8_/CdS heterojunction. This generates a strong electrochemical signal via broad-spectrum absorption and high charge separation efficiency. By polymerizing *m-*phenylenediamine, they constructed a customized 3D molecularly imprinted cavity for specific colchicine recognition, achieving ultrasensitive detection (Kui et al., 2025).

Beyond micro-level sensing, macroscopic clinical risk management requires in-depth pharmacovigilance data mining. Utilizing real-world spontaneous reporting databases for disproportionality analysis provides crucial large-sample warnings for unexpected adverse events in complex targeted or immune combinations. Ultimately, clinical decisions must adhere to local labels and guidelines: co-administering strong CYP3A4/P-gp inhibitors is strictly contraindicated in hepatic and renal impairment. Furthermore, clinical pharmacists must conduct comprehensive safety assessments before adjusting any multi-drug regimen.

## 5 Clinical translational challenges and critical analysis

Despite significant progress in structural modification, formulation optimization, and clinical management, colchicine’s modern anti-tumor translational research still faces a formidable “bench-to-bedside” gap. Objectively examining the current landscape reveals three core limitations that urgently require resolution.

First, within medicinal chemistry and structural modification, a significant disconnect exists between high *in vitro* activity and *in vivo* druggability. Colchicine’s SAR is rigidly constrained by the A- and C-rings, which dictate high-affinity tubulin binding but offer extremely narrow modification spaces. While the B-ring permits greater flexibility, universal optimization rules regarding steric hindrance and polarity remain elusive. Furthermore, despite exhibiting exceptional picomolar-to-nanomolar IC_50_ values *in vitro*, most novel multi-site derivatives lack systematic ADMET (absorption, distribution, metabolism, excretion, and toxicity) profiling. Alarmingly, the SI of certain derivatives remains ≤1, indicating uncontrolled off-target toxicity. This unresolved “narrow therapeutic window” traps most candidates in early preclinical cell screening or mouse models, severely hindering their substantive clinical advancement (Rubicondo et al., 2025).

Second, concerning formulation innovation and targeted delivery, an over-reliance on the EPR effect obscures the true difficulties of solid tumor penetration. An authoritative meta-analysis revealed that the median nanoparticle delivery efficiency to solid tumors is a mere 0.7% of the injected dose. Consequently, most colchicine nanoformulations fail to reach the tumor target and are instead intercepted by the mononuclear phagocyte system, accumulating off-target in vital organs like the liver and spleen. This inefficiency drastically diminishes expected efficacy and exponentially increases systemic toxicity risks. Moreover, current delivery technologies predominantly remain at the proof-of-concept stage, severely lacking rigorous toxicological validation, robust scale-up processes, and substantive clinical translational validation (Ghawanmeh et al., 2020; Lei et al., 2024; Wilhelm et al., 2016).

Finally, regarding clinical management and DDI assessment, current risk warning systems severely lack evidence-based support from real-world oncology cohorts. When evaluating colchicine combinations with cutting-edge targeted therapies (e.g., TKIs and CDK4/6 inhibitors), studies rely heavily on theoretical *in vitro* pharmacological deductions. Clinical guidelines often mechanically extrapolate PK data from non-tumor cohorts, such as gout patients. However, tumor-induced systemic inflammation and massive pro-inflammatory cytokine release significantly downregulate baseline hepatic CYP3A4 expression, drastically lowering the metabolic tolerance threshold of oncology patients compared to the general population (Harvey and Morgan, 2014). Relying on single-mechanism deductions without robust validation from real-world PK/PD data is clinically inadequate, risking unpredictable and fatal toxicities in complex multi-line regimens and failing to guide precise individualized medication.

## 6 Conclusions and perspectives

As a classical microtubule-targeting agent (MTA), colchicine holds profound potential for targeted anti-tumor intervention via the CBS. However, bridging the translational gap to truly realize its value in precision oncology demands a relentless focus on “toxicity reduction and efficacy enhancement” across three frontier directions. First, in medicinal chemistry, future molecular design must transcend traditional “competitive occupancy” models toward “multidimensional synergistic” strategies. Alongside finely mapping binding thermodynamics, researchers should explore targeted protein degradation (TPD) technologies, such as PROTACs, that couple optimized low-toxicity pharmacophores with the ubiquitin-proteasome system. This “classical scaffold + targeted degradation” chimera could specifically induce complete tubulin degradation, fundamentally circumventing the systemic toxicity driven by high-dose or long-term traditional inhibition (Assunçao et al., 2025). Second, in formulation optimization and targeted delivery, next-generation nanocarriers and antibody-drug conjugates (ADCs) must evolve toward dynamic intelligence. R&D should focus on exploiting the unique TME—such as hypoxia, mild acidity, and specific enzyme overexpression—to engineer highly sensitive, stimuli-responsive smart nanocarriers. These systems can overcome physical and stromal barriers to achieve deep tumor penetration and execute spatiotemporally “on-demand” drug release, thereby substantially widening the therapeutic index (Mura et al., 2013). Finally, to fortify clinical pharmacovigilance and DDI management, novel ultrasensitive sensing technologies (e.g., molecularly imprinted photoelectrochemical sensors) must be rapidly translated into routine TDM tools. Concurrently, it is imperative to construct patient stratification strategies grounded in PK/PD mathematical modeling and real-world biomarkers. This approach will facilitate precise, real-time individualized dose interventions for patients navigating complex multi-line combination therapies. Ultimately, through the deep cross-integration of medicinal chemistry, materials science, and clinical pharmacology to comprehensively shatter its druggability barriers in activity, delivery, and metabolism, this ancient alkaloid is poised to be revitalized and reshaped into an indispensable, heavyweight asset within the modern precision oncology arsenal.
